# Proteomic Signatures Involved in Cardiac Recovery After Mechanical Unloading

**DOI:** 10.1161/CIRCULATIONAHA.124.073093

**Published:** 2025-11-25

**Authors:** Yukako Shintani-Domoto, Koji L. Ode, Seitaro Nomura, Yoshiki Nagashima, Osamu Kinoshita, Manami Katoh, Takanobu Yamada, Eisuke Amiya, Kanna Fujita, Hiroyuki Abe, Tetsuo Ushiku, Issei Komuro, Minoru Ono, Hiroki R. Ueda, Masashi Fukayama

**Affiliations:** 1 Department of Pathology (Y.S.-D., H.A., T.U., M.F.), University of Tokyo Hospital, University of Tokyo, Japan.; 2 Department of Systems Pharmacology (K.L.O., Y.N., H.R.U.), University of Tokyo Hospital, University of Tokyo, Japan.; 3 Department of Cardiovascular Medicine (S.N., M.K., T.Y., E.A., K.F., I.K.), University of Tokyo Hospital, University of Tokyo, Japan.; 4 Department of Frontier Cardiovascular Science (S.N., M.K., I.K.), University of Tokyo Hospital, University of Tokyo, Japan.; 5 Department of Computational Diagnostic Radiology and Preventive Medicine (K.F.), University of Tokyo Hospital, University of Tokyo, Japan.; 6 Department of Cardiothoracic Surgery (O.K., M.O.), University of Tokyo Hospital, University of Tokyo, Japan.; 7 International University of Health and Welfare, Japan (I.K.).; 8 Department of Integrated Diagnostic Pathology, Nippon Medical School, Tokyo, Japan (Y.S.-D.).; 9 Thermo Fisher Scientific, Inc, Yokohama, Japan (Y.N.).; 10 Department of Cardiovascular Surgery, Saitama Medical University, Japan (O.K.).; 11 Laboratory for Synthetic Biology, RIKEN Center for Biosystems Dynamics Research, Suita, Japan (H.R.U.).; 12 Department of Systems Biology, Institute of Life Science, Kurume University, Japan (H.R.U.).; 13 Asahi TelePathology Center, Asahi General Hospital, Japan (M.F.).

**Keywords:** heart failure, prognosis, proteomics

Heart failure is a leading cause of death worldwide, and patients with severe heart failure require left ventricular assist device (LVAD) support.^1^ LVAD is usually implanted as a bridge to transplantation (BTT) or destination but can be removed if the patient’s cardiac function recovers. However, the molecular predictors for cardiac prognosis after LVAD implantation remain elusive.

We performed liquid chromatography–mass spectrometry on myocardial samples from heart transplant recipients, including 14 BTT cases and 10 bridge to recovery (BTR) cases, excluding patients with myocarditis. This study was approved by the institutional review board of the University of Tokyo (No. 10162, G10032). The data that support the findings of this study are available from the first authors upon reasonable request. LVAD implantation duration averaged 849 days (range, 68–1,405) in BTT and 202.8 days (range, 77–428) in BTR (Figure [A]). Myocardial tissues were processed, and formalin-fixed and paraffin-embedded specimens were analyzed. Samples from BTT were collected at both LVAD implantation (pre-LVAD) and heart transplantation (post-LVAD), whereas BTR samples were collected only at LVAD implantation. The samples were analyzed by data-dependent tandem mass spectrometry with a mass spectrometer (Q-Exactive Mass Spectrometer, Thermo Fisher Scientific). Mass spectrometry data were analyzed and quantified using Proteome Discoverer version 1.4 (Thermo Fisher Scientific) with the Swiss-Prot section of the UniProtKB human database (as of May 28, 2014). Only proteins that were consistently quantified in at least 11 of 14 BTT patients and 8 out of 10 BTR patients were included in the statistical analysis.

**Figure.**
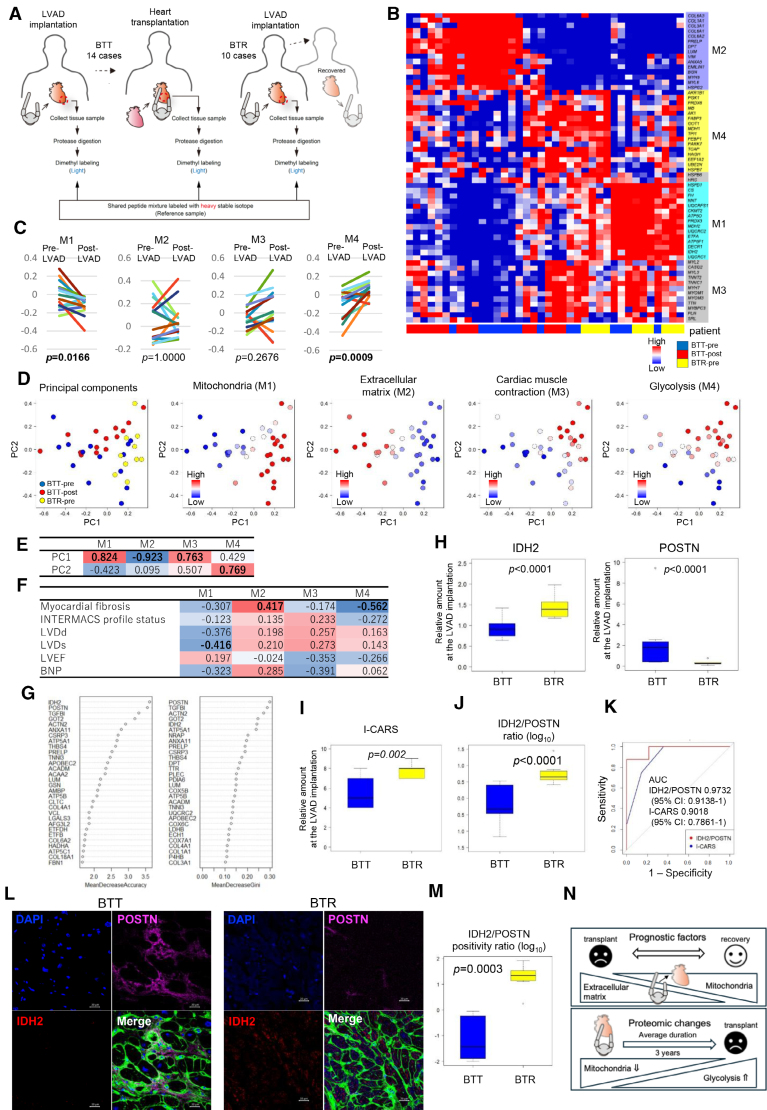
**Proteomic signatures involved in cardiac recovery after mechanical unloading. A**, Scheme of the study design for the proteomic analysis. In the bridge to transplant (BTT) group (14 patients [11 male, 3 female]; mean age, 41.3 years; range, 19–55) and the bridge to recovery (BTR) group (10 patients [7 male, 3 female]; mean age, 34 years; range, 21–43), tryptic peptides were obtained from pre-LVAD samples collected from the left ventricular apex at LVAD implantation. In the BTT group, additional post-LVAD samples were collected from the left ventricular free wall at heart transplantation. All peptides were covalently labeled with a light methyl (CH₃) tag. A mixture of all samples of BTT and BTR was labeled with heavy label (CHD_2_), enabling relative quantification at each time point. There was no statistically significant difference in age between the BTT and BTR groups (*P*=0.098). **B**, Hierarchical clustering performed with pre- and post-LVAD of BTT and pre-LVAD of BTR. Weighted Gene Coexpression Network Analysis (WGCNA) identified 4 modules (M1–M4). Each cluster is characterized as follows: M1 represents mitochondria, M2 represents the extracellular matrix, M3 represents cardiac muscle contraction, and M4 represents glycolysis. **C**, Protein changes of BTT in each module. During LVAD support, the expression of M4 was elevated (Wilcoxon signed-rank test, *P*=0.0009), whereas that of M1 was decreased (Wilcoxon signed-rank test, *P*=0.0166). **D**, Principal component analysis (PCA). The **left** plot shows the samples plotted according to their PCA scores on PC1 and PC2, labeled by sample origin (BTT-pre, BTT-post, BTR-pre). In the adjacent plots for M1 to M4, the positions of the samples on PC1 and PC2 are retained, whereas the samples are labeled according to the module eigengene (ME) expression values for each module. PCA indicated that PC1 contributed to the distinction between BTT and BTR at the time of LVAD implantation, whereas PC2 reflected changes before and after LVAD implantation in BTT. **E**, Correlation coefficient analysis between PCA and each module. **F**, The correlation coefficient between module expressions and clinicopathological data at the time of LVAD implantation, including myocardial fibrosis, Interagency Registry for Mechanically Assisted Circulatory Support (INTERMACS) profile status, left ventricular end-diastolic dimension (LVDd), left ventricular end-systolic dimension (LVDs), left ventricular ejection fraction (LVEF), and BNP (brain natriuretic peptide). The analysis revealed that LVDs negatively correlated with M1, whereas myocardial fibrosis showed a negative correlation with M4 and a positive correlation with M2. **G**, The prognostic proteins selected by random forest analysis. IDH2 (isocitrate dehydrogenase [nicotinamide adenine dinucleotide phosphate, oxidized form] mitochondrial) and POSTN (periostin) were effective in separating the good prognosis group (BTR) from the poor prognosis group (BTT) at the time of LVAD implantation. **H**, Relative amount of IDH2 and POSTN at the time of LVAD implantation in BTT (n=14) and BTR (n=10). IDH2 (M1) was high in BTR, and POSTN (M2) was high in BTT. **I**, INTERMACS Cardiac Recovery Score (I-CARS) at the time of LVAD implantation was predominantly higher in BTR than in BTT (Mann-Whitney U test, *P*=0.002). **J**, Relative amount of IDH2/POSTN ratio at the time of LVAD implantation in BTT and BTR. IDH2/POSTN was high in BTR. **K**, The AUC of the receiver operating characteristic (ROC) curve for IDH2/POSTN was 0.9732 (95% CI, 0.9138–1.000), and that for I-CARS was 0.9018 (95% CI, 0.7861–1 [DeLong]). The comparison using the DeLong test showed that the difference in AUCs was not statistically significant (Z=1.1043; *P*=0.2695), but the area of ROC demonstrated that IDH2/POSTN ratio has better performance for predicting cardiac recovery compared with I-CARS. **L**, Representative images of anti-IDH2 (mouse monoclonal antibody; 200×; Abcam) and anti-POSTN (rabbit monoclonal antibody; 200×; ab227049) fluorescent double staining in myocardial tissue of another group (6 cases of BTT and 7 cases of BTR). The positive areas quantified under immunofluorescent staining of IDH2 (red) and POSTN (pink) were significantly different between the 2 groups. IDH2 was localized in the cytoplasm of cardiomyocytes and POSTN was localized in the fibrotic regions of the interstitium. **M**, The ratio of positive areas of IDH2 and POSTN was significantly higher in BTR group (Mann-Whitney U test, *P*=0.003). This powerful prognostic method was able to separate the samples into 2 groups, BTT and BTR, using a cutoff value of 0. The vertical axis is scaled in log_10_. **N**, Schematic summary of the study. Proteins involved in glycolysis gradually increased and mitochondrial proteins decreased after LVAD implantation. Cardiac reversibility was associated with more mitochondrial proteins and less extracellular matrix proteins at LVAD implantation.

We performed weighted gene coexpression network analysis using proteins that satisfied the above criteria among those detected by liquid chromatography–mass spectrometry in the myocardium of BTT and BTR (n = 412) and identified 4 modules (M1–M4): M1 (mitochondria-related, 149 proteins), M2 (extracellular matrix, 100 proteins), M3 (cardiac muscle contraction, 33 proteins), and M4 (glycolysis, 23 proteins). We applied expression profiles of module eigengenes to hierarchical clustering and showed that high module eigengene expression for M1 and low module eigengene expression for M2 were characteristic of BTR at LVAD implantation (Figure [B]). During LVAD support in BTT, the expression of M4 was elevated, whereas that of M1 was decreased (Figure [C]).

Principal component (PC) analysis indicated that PC1 contributed to the distinction between BTT and BTR at the time of LVAD implantation, whereas PC2 contributed to the change before and after LVAD implantation in BTT (Figure [D]). We calculated the correlation coefficient between PC analysis and each module eigengene expression (Figure [E]). PC1 showed a positive correlation with M1 and M3 and a negative correlation with M2. PC2 showed a positive correlation with M4. An analysis at LVAD implantation revealed negative correlations between left ventricular end-systolic dimension and M1, and myocardial fibrosis and M4, and a positive correlation between myocardial fibrosis and M2 (Figure [F]).

The random forest machine-learning algorithm was used to identify proteins that help distinguish between BTT and BTR (Figure [G]). Especially IDH2 (isocitrate dehydrogenase [nicotinamide adenine dinucleotide phosphate, oxidized form] mitochondrial) (M1) and POSTN (periostin) (M2) were identified as the most significant proteins involved in cardiac reversibility. Similar to the module expression profiles, the protein expression levels of IDH2 were high, whereas those of POSTN and TGFBI (transforming growth factor-β–induced protein) were low in the BTR group (Figure [H]). The Interagency Registry for Mechanically Assisted Circulatory Support (INTERMACS) Cardiac Recovery Score has been proposed as a predictor of prognosis after LVAD implantation. The INTERMACS Cardiac Recovery Score at the time of LVAD implantation was predominantly higher in BTR than in BTT (Figure [I]).^2^ Focusing on the IDH2/POSTN ratio as a better prognostic indicator, we demonstrated excellent discrimination of IDH2/POSTN for cardiac recovery based on the area under the receiver operating characteristic curve (Figure [J and K]).

Furthermore, we collected myocardial tissues of another cohort and performed immunofluorescent double staining for IDH2 and POSTN (Figure [L]). The ratio of IDH2/POSTN-positive areas was significantly different between the 2 groups (Figure [M]), validating the efficacy of IDH2/POSTN ratio by immunostaining for predicting the cardiac reversibility after LVAD.

The IDH2/POSTN ratio was identified as a powerful predictor for cardiac reversibility. IDH2 was known to play a pivotal role in the mitochondrial antioxidant pathway by generating nicotinamide adenine dinucleotide phosphate, reduced form and maintaining an appropriate redox balance. Mitochondrial dysfunction is associated with an imbalance in energy supply and demand for heart failure; its correction has attracted attention as a new therapeutic approach to improve overall cardiac function.^3^ POSTN has been recognized for its important role in extracellular matrix development.^4^ Decreased mitochondria and increased extracellular matrix may reflect a decline in cardiac function.

Glycolysis (M4) protein levels were increased after LVAD implantation in BTT. In heart failure, the myocardial energy metabolism is known to shift toward a fetal-like pattern, characterized by a decrease in fatty acid metabolism and an increase in glycolysis,^5^ suggesting the proteomic remodeling associated with metabolic changes after LVAD.

In conclusion, proteomic analysis of myocardial tissues before and after LVAD revealed significant protein changes and identified potential recovery markers. Although protein changes may be attributable to differences in sampling sites, the IDH2/POSTN ratio was useful to predict BTR, which will lead to the development of precision medicine for heart failure with LVAD.

## Article Information

### Acknowledgments

We thank K. Sakuma and A. Nishimoto for preparing samples; M. Makita, T. Kitamura, and Dr Kousuke Ishino for helping with proteomics sample preparation; I. Sakamoto for immunostaining; Dr. Yoshimasa Kawazoe for helping with statistical analysis; and Dr. Ryuji Ohashi for institutional support and guidance.

### Sources of Funding

This study was supported by the Japan Society for the Promotion of Science (No. 15K20942 to Y.S.-D., Nos. 22H00471 and JP25H01050 to S.N., No. 18H05270 to H.R.U., and Nos. JP21H05045 and JP24K23940 to I.K.), Japan Science and Technology Agency (JST) Exploratory Research for Advanced Technology (ERATO) (No. JPMJER1904 to H.R.U.), Human Frontier Science Program Research Grant Program (No. RGP0019/2018 to H.R.U.), UTEC-Utokyo FSI Research Grant Program (to S.N.), JST Fusion Oriented Research for Disruptive Science and Technology (FOREST) Program (No. JPMJFR210U to S.N.), Japan Agency for Medical Research and Development (AMED) (Nos. JP18km0405209, JP18gm6210010, JP21ek0109543, JP22ama121016, JP22ek0210172, JP22ek0210167, JP22bm1123011, JP23tm0724607, JP23gm4010020, JP223fa627011, JP22ek0109617, JP23tm0524009, JP23tm0524004, JP23jf0126003, JP24ek0109755, JP24ek0210205, JP24jf0126011, JP25bk0104192, and JP25ek0109795 to S.N. and I.K.), and Initiative for Realizing Diversity in the Research Environment from Ministry of Education, Culture, Sports, Science and Technology (MEXT), Japan (to Y.S.-D.).

### Disclosures

H.R.U. conducted a collaborative research project with Thermo Fisher Scientific, Inc. Y.N. was an employee of Thermo Fisher Scientific, Inc. The other authors report no conflicts.
